# Influence of Ru on structure and corrosion behavior of passive film on Ti-6Al-4V alloy in oil and gas exploration conditions

**DOI:** 10.1038/s41598-022-21047-0

**Published:** 2022-10-05

**Authors:** Qiang Liu, Hongtao Liu, Junfeng Xie, Wei-fu Zhang, Yi-ming Zhang, Chun Feng, Guang-shan Li, Yang Yu, Sheng-yin Song, Cheng-xian Yin

**Affiliations:** 1 State Key Laboratory for Performance and Structural Safety of Petroleum Tubular Goods and Equipment Materials, CNPC Tubular Goods Research Institute, No. 89, Jinye 2Nd Road, Xi’an, 710077 Shaanxi China; 2 Petroleum Engineering Institute, Tarim Oil Field Company of CNPC, Kuerle, 841000 Xinjiang China; 3 School of Materials Science and Engineering, Xi’an University of Technology, No.5 Jinhua South Road, Xi’an, 710048 China; 4 State Key Lab of Nonferrous Metals and Processes, GRINMAT Engineering Institute Co, Ltd., No. 11, Xingke East Street, Beijing, 101400 China

**Keywords:** Materials for energy and catalysis, Structural materials

## Abstract

In order to investigate the influence of minor Ru on the electrochemical behaviour and structural characteristics of passive films on the surface of Ti-6Al-4V alloys under various oil and gas exploration conditions, electrochemical techniques, X-ray photoelectron spectroscopy (XPS), scanning electron microscope (SEM) and corrosion simulation tests were carried out. The results revealed that the oil and gas exploration conditions had a serious impact on the electrochemical behaviour and corrosion resistance of the tested alloys. The passivation film resistance and corrosion potential of the tested titanium alloys were significantly reduced with increasing acidity and temperature. With the addition of minor ruthenium, the potential of the passive film on the Ti-6Al-4V-0.11Ru alloy surface increased because of the high surface potential of the ruthenium element. The contents of metallic ruthenium and tetravalent titanium oxide TiO_2_ in the surface film of the Ti-6Al-4V-0.11Ru alloy both increased with increasing temperature, which led to increase the thickness, stability, corrosion resistance and repairability of the passive film on the surface of the Ti-6Al-4V-0.11Ru alloy being better than those qualities of Ti-6Al-4V. These results were also confirmed by corrosion simulation tests.

## Introduction

With the development of oil and gas exploration in deep water and under high-temperature, high-pressure, high-corrosion and other unconventional oil and gas resource conditions, there is a massive demand for new oil country tubular goods (OCTG) materials with high corrosion resistance and high performance^1–4^. Titanium alloys have become attractive candidate materials for OCTG and offshore components in harsh service conditions, owing to their high specific strength, excellent corrosion resistance, long fatigue life and outstanding mechanical properties^5–9^. However, most of the common titanium alloys have been developed for aerospace, biomedicine, shipping and marine engineering applications, whereas there are large differences in the application environments of the abovementioned fields and oil and gas exploration^10–12^. Certain titanium alloys will undergo corrosion in oil and gas exploration environments, which will lead to drilling failure, completion strings and major economic losses^13,14^.

Many studies have been conducted to improve the corrosion resistance of titanium alloys under oil and gas exploration conditions. Kitayama^15^ studied the corrosion behaviour of titanium alloys in a sulfur-containing H_2_S-CO_2_-Cl^−^ environment and clarified that the addition of Pd and/or the increase in Mo content in titanium alloys was very effective for improving the SCC resistance. Tomashov et al.^16^ found that additions of Pd or Pt to titanium alloys increased their corrosion resistance towards more concentrated HCl and H_2_SO_4_ solutions. Sedricks^17^ found that the corrosion resistance of the binary titanium alloy Ti-Ru was comparable to that of Ti-Pd alloys in H_2_SO_4_, and the corrosion resistance was only slightly inferior in HCl media. Schutz^18–22^ proposed that additions of platinum group metals (PGMs) to existing commercial unalloyed titanium and alpha–beta alloys were shown to be crevice corrosion and stress corrosion resistant to sweet and sour hot, acidic NaCl-rich brines at high temperatures. van der Lingen^23^ indicated that titanium-ruthenium alloys formed a secondary phase and compared well with palladium-containing titanium alloys. However, there are harsh environments for oil and gas exploration in China. The underground pipe strings are not only confronted with the challenges of high temperatures and high pressures but they also suffer from impacts by hydrogen sulfide, carbon dioxide, high concentration saline/completion fluid, elemental sulfur and strong acid. Consequently, the demand for oil country tubular goods in China exceeds that of other countries. Furthermore, the difference in anti-corrosion properties and adaptability between the most widely used titanium alloy (Ti-6Al-4V) and PGM-containing titanium alloys in these service conditions is unknown, and the corrosion resistance and surface electrochemical behaviour of ruthenium-containing Ti-6Al-4V alloys in harsh oil and gas exploration environments are unclear.

Unfortunately, there has not been any literature reporting the effects of Ru on the corrosion behaviour and structural characteristics of passive films on Ti-6Al-4V alloys in such service conditions, and the lack of research results poses a risk in selecting and using titanium alloy OCTG for oil and gas exploration. Therefore, the purpose of this study is to investigate the difference in electrochemical behaviours, including open-circuit potentials, potentiodynamic polarization, and electrochemical impedance spectroscopy, as well as the corrosion resistance of Ru-containing titanium alloy in comparison with Ti-6Al-4V alloy in a harsh oil and gas exploration environment, with X-ray photoelectron spectroscopy (XPS) and scanning electron microscope (SEM) analyses. Finally, the influence of Ru on the characteristics and passivation mechanism of the passive film on the surface of titanium alloy are discussed.

## Results

### Open-circuit potential

**Figure 1:**
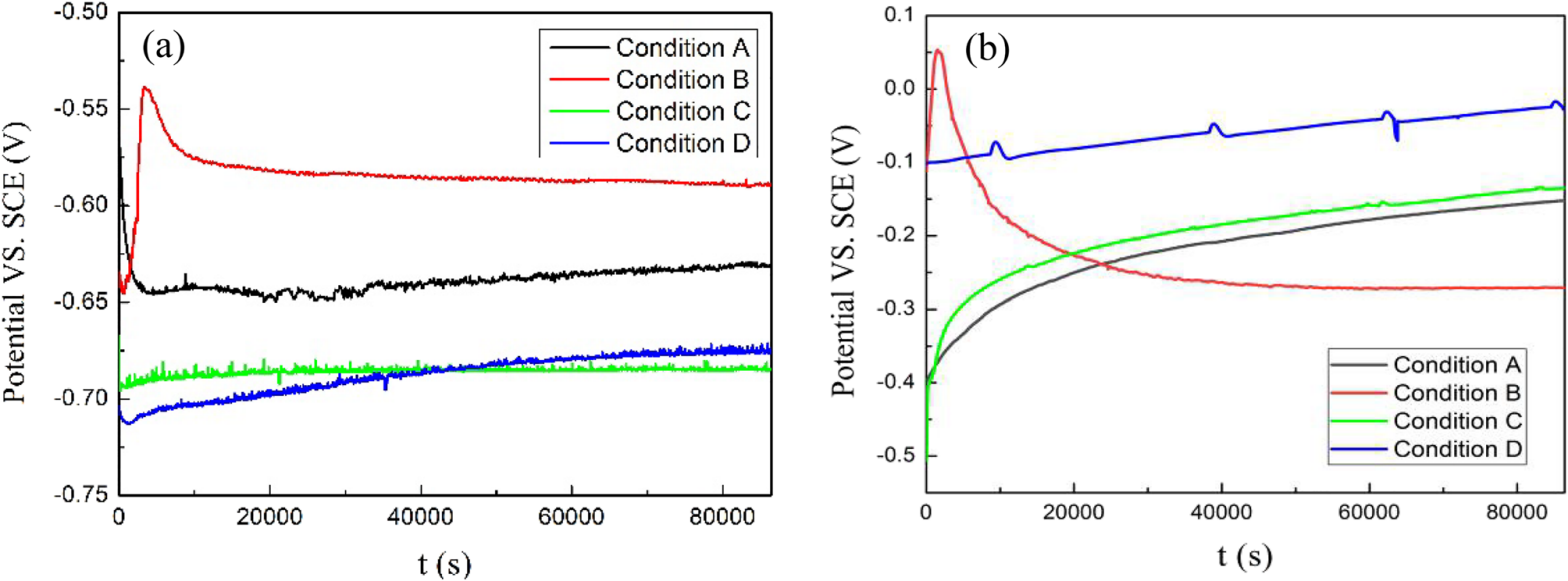
OCP evolution with immersion time for different titanium alloy samples under test conditions: (**a**) Ti-6Al-4V and (**b**) Ti-6Al-4V-0.11Ru.

The open-circuit potential can initially reflect the electrochemical behaviour at the electrode/solution interface, while the near-steady corrosion potential is determined by the electrochemical kinetics and thermodynamics and can reflect the passivation behaviour of metal in solution. The variations in OCP versus immersion time for Ti-6Al-4V and Ti-6Al-4V-0.11Ru are shown in Fig. 1a,b, respectively.

The OCPs of the two titanium alloy materials changed rapidly at the beginning of the immersion tests, but with increasing immersion time, the OCPs of the materials gradually reached a plateau, indicating that passive films were rapidly formed and stabilized on the surfaces of the titanium alloys under these conditions^24–27^. It can be clearly observed from Fig. 3 that the final OCP values of the Ti-6Al-4V-0.11Ru alloy were substantially more positive than those of the Ti-6Al-4V alloy under the test conditions. This significant difference in the OCP values indicates that the addition of Ru can dramatically increase the potential of the Ti-6Al-4V alloy surface. The final OCP values of the Ti-6Al-4V alloy and the Ti-6Al-4V-0.11Ru alloy stabilized at approximately − 632 mV_SCE_ and − 152 mV_SCE_, respectively, at 23 °C when pH = 3, and the steady state OCP of the Ti-6Al-4V alloy shifted positively while the OCP of the Ti-6Al-4V-0.11Ru alloy moved in the opposite direction with increasing acidity at the same temperature, 23 °C. However, when the temperature increased under the same acidic conditions (pH = 1.5), the potential changes of the two alloys showed different passivation characteristics. The OCP of the Ti-6Al-4V alloy dropped from − 589 mV_SCE_ at 23 °C to − 685 mV_SCE_ at 60 °C and increased to − 676 mV_SCE_ when the temperature further increased to 100 °C, indicating that the stability of the passive film decreased with increasing temperature. In contrast, the OCP value of the Ti-6Al-4V-0.11Ru alloy shifted significantly from − 271 mV_SCE_ to − 27 mV_SCE_ when the test temperature increased from 23 to 100 °C, revealing that the thermodynamic stability of the Ti-6Al-4V-0.11Ru alloy was better than that of Ti-6Al-4V^28^.

### Potentiodynamic polarization

**Figure 2:**
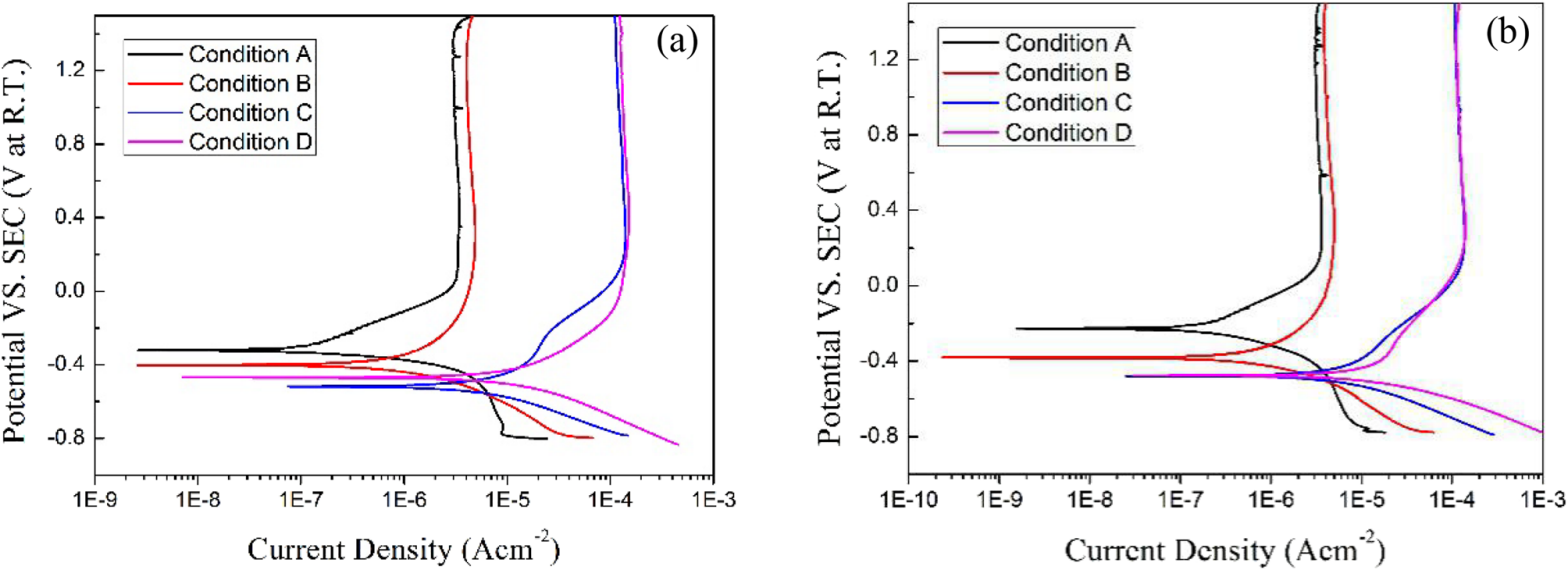
Potentiodynamic polarization curves for the tested titanium alloy samples under various test conditions: (**a**) Ti-6Al-4V and (**b**) Ti-6Al-4V-0.11Ru.

Figure 2 shows the potentiodynamic polarization curves of Ti-6Al-4V and Ti-6Al-4V-0.11Ru alloy under different test conditions after immersion in solution for 72 h. The electrochemical parameters were determined and summarized in Table 1. The corrosion potential (E_corr_) was obtained at the potential of current change from cathodic to anodic. Figure 2 indicates that there was a clear passivity domain in the potentiodynamic polarization curves of Ti-6Al-4V and Ti-6Al-4V-0.11Ru alloy under different test conditions, and this domain continued at more anodic potentials. Thus, the corrosion current, I_corr_, was equal to the passive current, I_PASS_, which was obtained at 0.3 V_SCE._

**Table 1:** Electrochemical parameters of different titanium alloys under various test conditions.

Condition/materials		E_corr_/mV	I_corr_/A cm^−2^	Bc/mVdec^−1^
Con. A	Ti-6Al-4V	− 359	3.766 × 10^–6^	− 61.06
	Ti-6Al-4V-0.11Ru	− 262	3.220 × 10^–6^	− 137.67
Con. B	Ti-6Al-4V	− 397	4.997 × 10^–6^	− 224.06
	Ti-6Al-4V-0.11Ru	− 335	4.677 × 10^–6^	− 190.15
Con. C	Ti-6Al-4V	− 475	1.604 × 10^–4^	− 75.58
	Ti-6Al-4V-0.11Ru	− 434	1.372 × 10^–4^	− 66.69
Con. D	Ti-6Al-4V	− 425	1.692 × 10^–4^	− 102.07
	Ti-6Al-4V-0.11Ru	− 413	1.401 × 10^–4^	− 74.00

It can be seen from the figure that when the potential of the anodic polarization branch of the two titanium alloys exceeded 0 V_SCE_ under all test conditions, the corrosion current densities remained basically stable, indicating that obvious passivation zones formed on the surface of the tested alloys under different test conditions. In this passive region, the electrochemical corrosion reactions were all controlled by anodic reactions, and both titanium alloys generated passive films in all simulation solutions. With increasing acidity and temperature, the passivation films required higher passivation current densities to remain stable, as shown in Fig. 2.

Comparing the potentiodynamic polarization curves of the two titanium alloys, the corrosion potential of both titanium alloys both shifted negatively with decreasing pH values at the same test temperature, while the corrosion current density increased significantly. The I_corr_ of the Ti-6Al-4V alloy was almost the same as that of the Ti-6Al-4V-0.11Ru alloy when the pH was equal to 1.5 at 23 °C. However, the two titanium alloys showed different characteristics as the acidity remained constant (pH = 1.5) and the test temperature increased. The corrosion potential of the Ti-6Al-4V alloy decreased significantly, and the corrosion current showed a slight decrease and then a rapid increase, as shown in Fig. 2a, indicating that the temperature had a great influence on the corrosion resistance of the Ti-6Al-4V alloy. The Ti-6Al-4V alloy has a corrosion resistance threshold of approximately 60 to − 80 °C, which was in good agreement with the previous study results of Schuzt et al.^29^ The corrosion potential of the Ti-6Al-4V-0.11Ru alloy decreased and then stabilized at approximately − 413 mV_SCE_ with increasing temperature, as shown in Fig. 2b.

**Figure 3:**
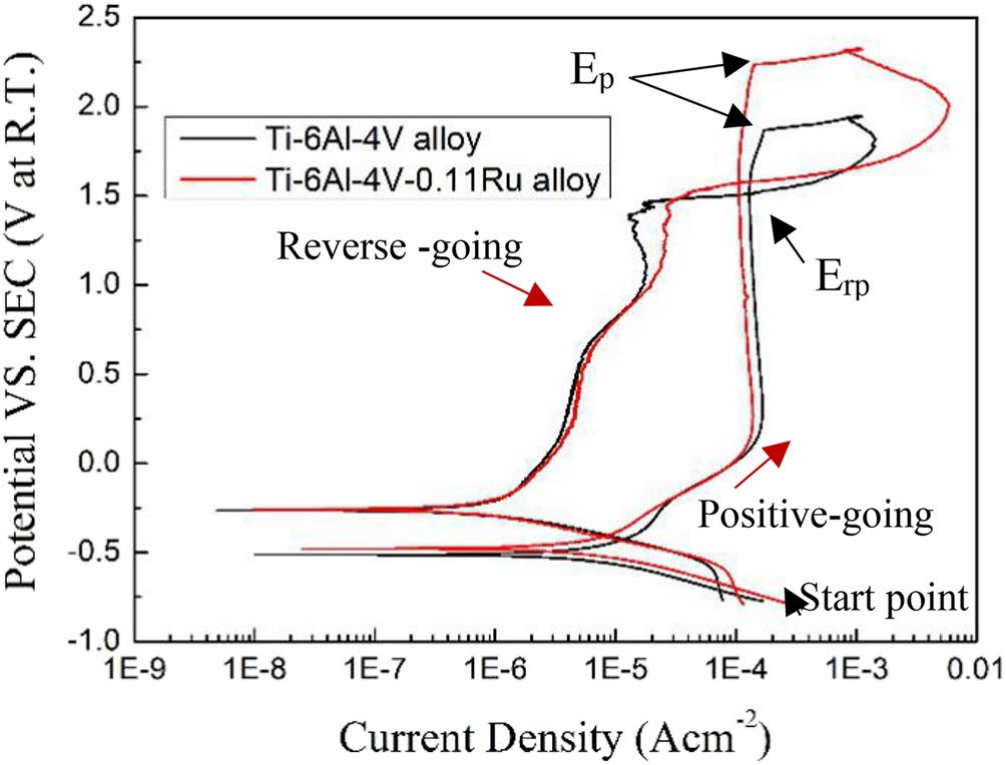
Cyclic polarization curves of different titanium alloys under test Condition C.

Figure 3 illustrates the cyclic potentiodynamic polarization test results of Ti-6Al-4V and Ti-6Al-4V-0.11Ru alloy in Condition C. The reversed polarization curves of the two titanium alloys intersected with the polarization curves in the anodic branch region, indicating that both tested titanium alloys had good passivation and repassivation ability under the test conditions. Cao’s work^30^ showed that the repairability of the passive film depended on the thickness of the original passive film, the protection potential E_rp_, and the pitting potential E_p_ of the material. The higher the protection potential E_rp_ was, the stronger the repassivation tendency of the material surface would be, and the pitting potential E_p_ was correlated with the repairability of the surface pitting corrosion. It could be derived from Fig. 3 that the pitting potential E_p_ of Ti-6Al-4V-0.11Ru alloy was approximately 2237 mV_SCE_ in Condition C, which was higher than the 1868mV_SCE_ of Ti-6Al-4V alloy and exhibited better resistance to surface pitting corrosion. The protective potential E_rp_ of the two titanium alloys showed insignificant differences under the test conditions, the E_rp_ of Ti-6Al-4V alloy and Ti-6Al-4V-0.11Ru alloy were 1572mV_SCE_ and 1512mV_SCE_, respectively, which indicating the similar repassivation of the surface film.

### Electrochemical impedance spectroscopy

**Figure 4:**
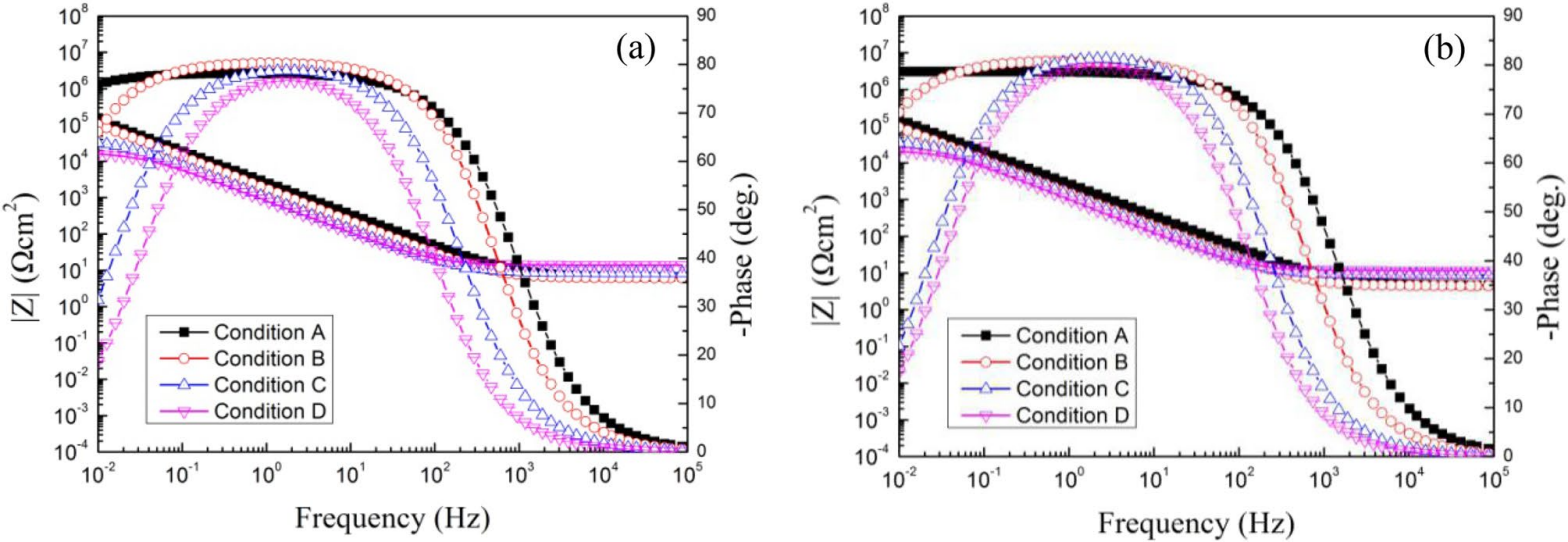
Bode diagrams for tested titanium alloy samples under various test conditions: (**a**) Ti-6Al-4V and (**b**) Ti-6Al-4V-0.11Ru.

To further study the influence of ruthenium on the electrochemical characteristics and passive film thickness of the Ti-6Al-4V alloy under different test conditions, EIS measurements were performed for the Ti-6Al-4V alloy and Ti-6Al-4V-0.11Ru alloy after the OCP test. Figure 4 shows the Bode diagrams of the two titanium alloys under different conditions. It was revealed that the phase angles of both tested titanium alloys had only one wide obvious peak from the low frequency to the high frequency, indicating that the impedance had one time constant. The maximum phase angles of both titanium materials were close to 80°, which was indicative of typical capacitive characteristics. With the addition of Ru in the Ti-6Al-4V alloy, the frequency range of obtaining a high, relatively constant phase angle and the phase angle maximum all increased under the same test conditions, indicating the difference between the Ti-6Al-4V alloy and Ti-6Al-4V-0.11Ru alloy in the solid/liquid interface, particularly under high-temperature conditions. The absolute impedance curves of the two titanium alloys showed similar behaviour under the same test conditions. The absolute impedance was independent of frequency in the high frequency regime and increased with almost the same slope from high frequency (10^3^ Hz) to low frequency (10^–2^ Hz) values. In the low-frequency regime, the absolute impedance decreased with increasing acidity and temperature, illustrating that ambient temperature and acidity have a significant influence on the characteristics of passive films on the alloy surface, as shown in Fig. 4.

**Figure 5:**
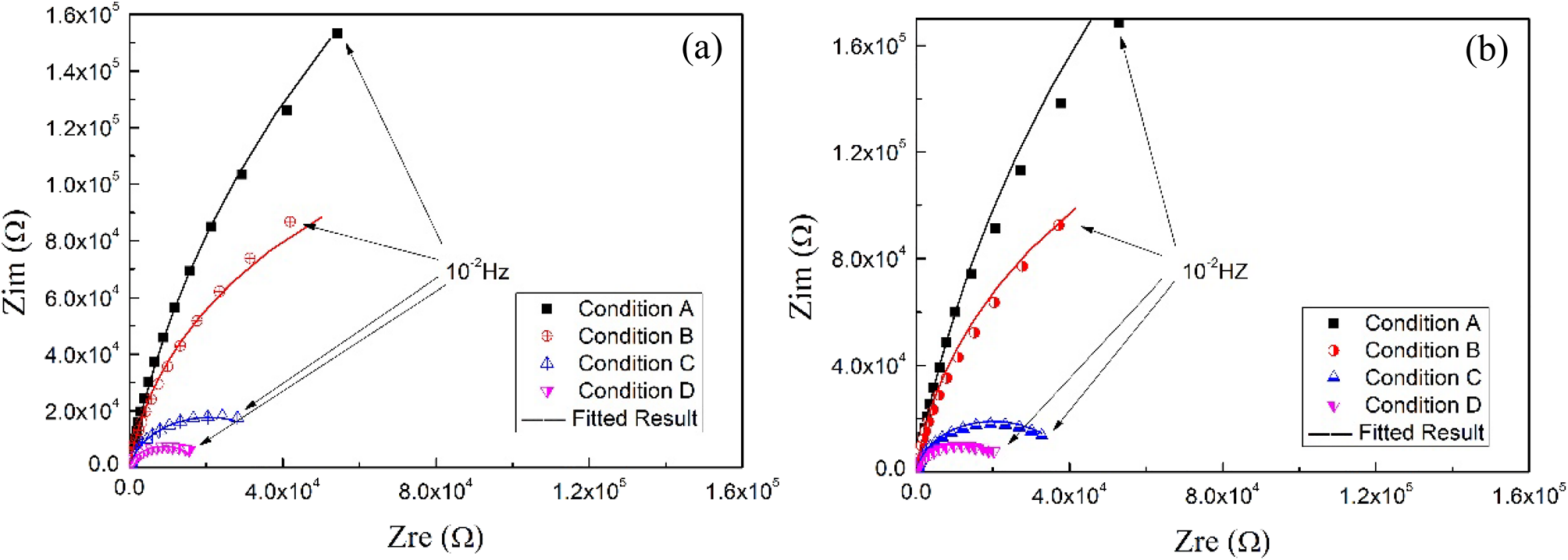
Nyquist diagrams for tested titanium alloy samples under various test conditions: (**a**) Ti-6Al-4V and (**b**) Ti-6Al-4V-0.11Ru.

**Figure 6:**
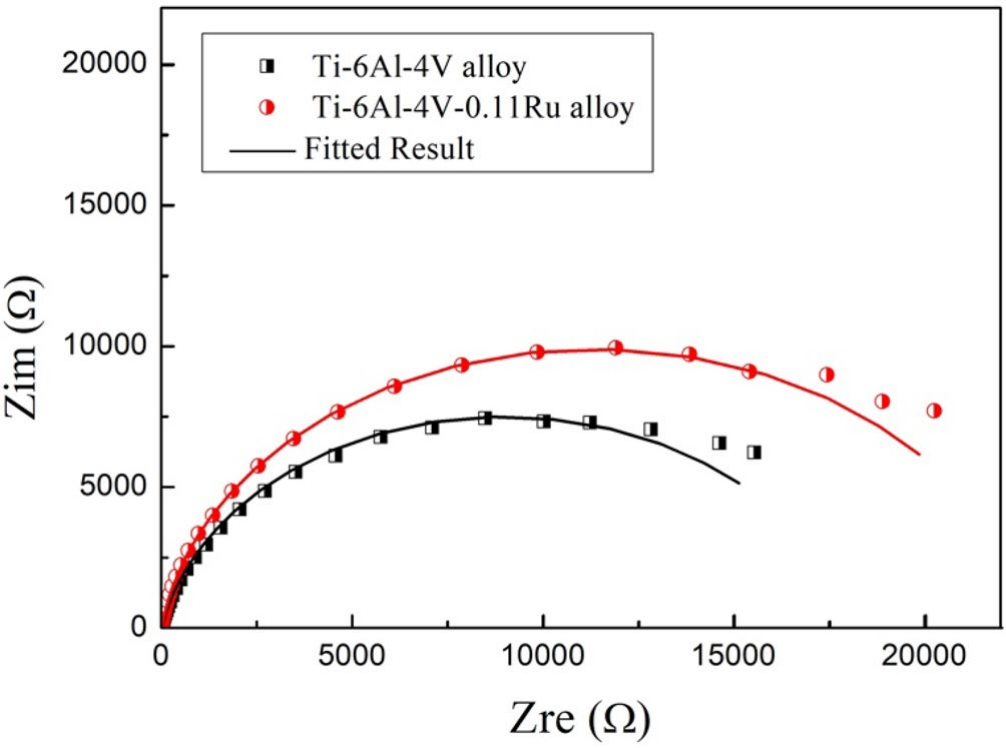
Nyquist diagrams for tested titanium alloy samples under Condition D.

Figure 5 illustrates the Nyquist plots of the two titanium alloys under different test conditions. From the diagrams, it can be seen that the two Nyquist plots showed the same characteristics; they both displayed a large semicircle capacitive loop with a time constant in both alloys, and the diameter of the capacitive loop was reduced with increasing acidity and temperature. When the temperature reached 100 °C, the loops of both titanium alloys were drastically smaller. By comparing the Nyquist plots of the two tested alloys, it was observed that the capacitive loop of Ti-6Al-4V alloys without the element Ru was smaller, which was indicative of less corrosion resistance in the Ti-6Al-4V alloy than that of the Ti-6Al-4V-0.11Ru alloy, especially at high temperatures, as shown in Fig. 6.

**Figure 7:**
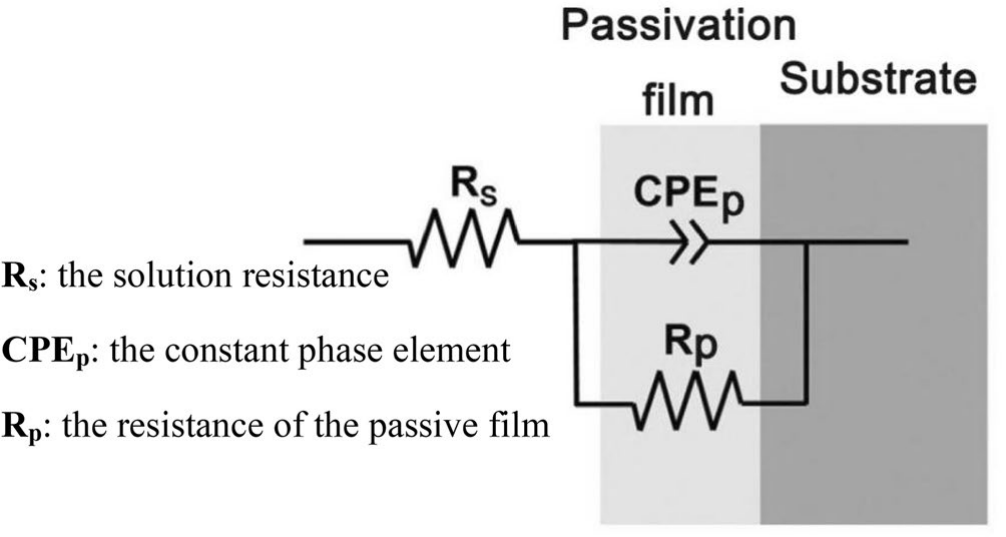
Equivalent electrical circuit model used for impedance spectra analysis of titanium alloys.

The EIS results of the two tested titanium alloys are in line with the most commonly used equivalent circuit model of dense passive films, and simplified Randles equivalent circuits were selected to fit the data^31–33^, as shown in Fig. 7, where R_s_ represents the solution resistance and R_p_ represents the resistance of the compact passivation film. The constant phase element (CPE_p_) was selected instead of pure capacitance due to the nonuniform distribution of current on the surface. CPE_p_ can be expressed as^25^:

1 $${\mathrm{Z}}_{{CPE}_{P}}=\frac{1}{Q{\left(j\omega \right)}^{n}}$$

where Q is the capacitance of the dense passive film, j is the imaginary unit, $\omega$ is the angular frequency and n is the deviation parameter, which is related to the constant phase angle^34^. When the value of n approaches 1, the phase angle constant CPE_p_ represents the dielectric layer formed on the surfaces of the solution and the alloy, and it behaves as the ideal capacitance Q. Based on the equivalent circuits model in Fig. 7, the impedance of the electrode and the resistance R_p_ can be calculated as follows:

2 $${Z}_{\omega }=\frac{1}{\frac{1}{{R}_{s}}+Q{\left(j\omega \right)}^{n}}$$

3 $${R}_{P}=\underset{\omega \to 0}{\mathrm{lim}}({Z}_{\omega })$$

The EIS fitting results are summarized and listed in Table 2. The deviation parameter n of the two tested titanium alloys was close to 1, so the phase angle constant CPE_p_ acted as an ideal parallel-plate capacitor Q_p_. The solution resistance R_s_ of the two alloys was almost the same under the same test conditions. However, the passivation film resistance R_p_ of all tested alloys was drastically reduced with increasing acidity and temperature. Under the same experimental conditions, the Ru-containing Ti-6Al-4V alloys exhibited a higher resistance R_p_ value than Ti-6Al-4V alloys, which meant that Ti-6Al-4V-0.11Ru had better corrosion resistance.

**Table 2:** Electrochemical parameters of different titanium alloys under various test conditions.

Items		R_s_/Ωcm^2^	R_p_/kΩcm^2^	Q_p_/Fcm^−2^s^n-1^	n
Con. A	Ti-6Al-4V	6.11 ± 0.24	816.04 ± 35.81	7.44 × 10^–5^ ± 0.00	0.913 ± 0.00
	Ti-6Al-4V-0.11Ru	4.53 ± 0.25	1011.30 ± 64.00	7.12 × 10^–5^ ± 0.00	0.919 ± 0.00
Con. B	Ti-6Al-4V	2.80 ± 0.19	257.05 ± 9.15	1.17 × 10^–4^ ± 0.00	0.929 ± 0.00
	Ti-6Al-4V-0.11Ru	3.08 ± 0.13	321.91 ± 6.92	1.19 × 10^–4^ ± 0.00	0.936 ± 0.00
Con. C	Ti-6Al-4V	4.85 ± 0.14	41.11 ± 0.39	1.90 × 10^–4^ ± 0.00	0.906 ± 0.01
	Ti-6Al-4V-0.11Ru	5.84 ± 0.11	40.47 ± 2.22	1.35 × 10^–4^ ± 0.00	0.941 ± 0.01
Con. D	Ti-6Al-4V	13.78 ± 0.20	17.95 ± 0.67	2.39 × 10^–4^ ± 0.00	0.886 ± 0.00
	Ti-6Al-4V-0.11Ru	11.69 ± 0.13	22.80 ± 0.17	1.77 × 10^–4^ ± 0.00	0.923 ± 0.01

To further study the passivation film behaviour of experimental alloys under various test conditions, with the assumption that the capacitance acts like a parallel-plate capacitor, the thickness of the passive film can be calculated according to the research of Xi and Wang et al.^25,31,35^,

4 $$\mathrm{C}=\frac{\varepsilon {\varepsilon }_{O}A}{d}$$

where $d$ is the thickness of the passive film, $\varepsilon$ represents the relative permittivity and is set to 48 for titanium^36^, ${\varepsilon }_{O}$ is the vacuum permittivity and is approximately 8.85 × 10^–14^ F cm^−1^, A is the effective surface area and is usually 3 times the geometric surface in cm^2^, and C represents the capacitance of the passive film. According to the work of Mark Orazem^37–39^, when a normal time constant is distributed through a surface layer, the relationship between the CPE_p_ parameters and effective capacitance requires an assessment of the characteristic time constant corresponding to the impedance of the layer. Therefore, the effective capacitance value C can be calculated from the CPE value from the following expression:

5 $$\mathrm{C}={{Q}_{p}}^{1/n}{{R}_{p}}^{(1-\mathrm{n})/n}$$

**Figure 8:**
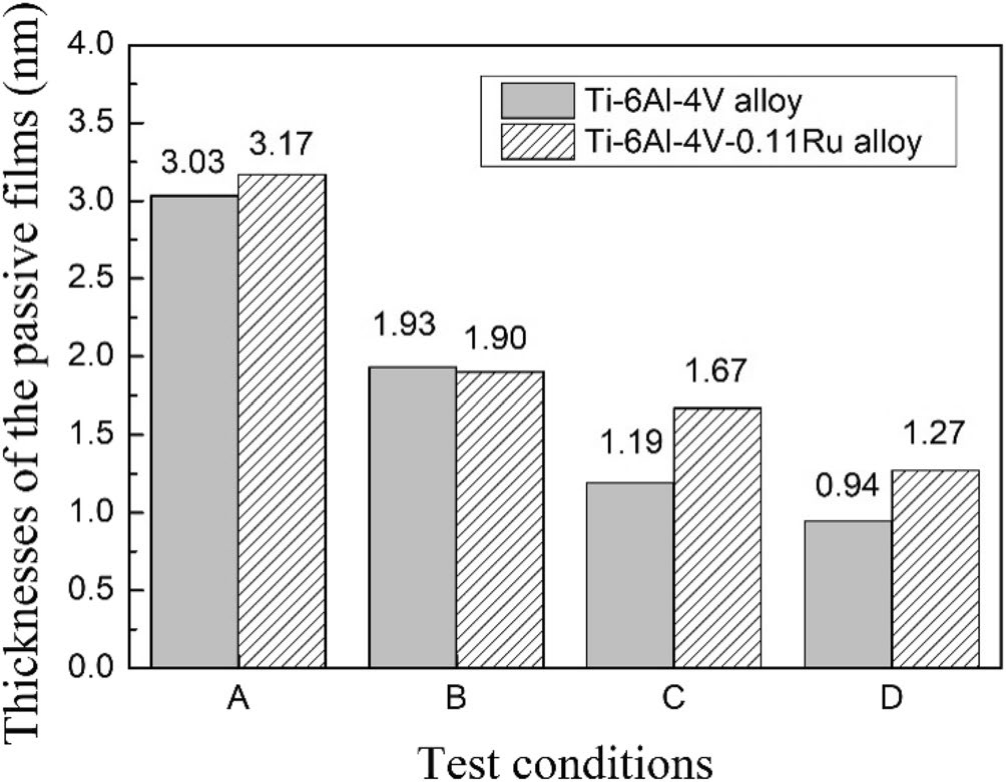
Thickness of the passive films for two alloys under different conditions.

Thus, the film thickness of the two tested alloys under various experimental conditions can be calculated and shown in Fig. 8. The average thickness of the passivation film on the Ti-6Al-4V-0.11Ru alloy surface was thicker than that of the Ti-6Al-4V alloy under different test conditions. The passivation film of the Ti-6Al-4V-0.11Ru alloy appeared much thinner when the temperature increased, which meant that the Ti-6Al-4V-0.11Ru alloy had better corrosion resistance in a harsh environment.

### Corrosion test and surface XPS analysis

**Figure 9:**
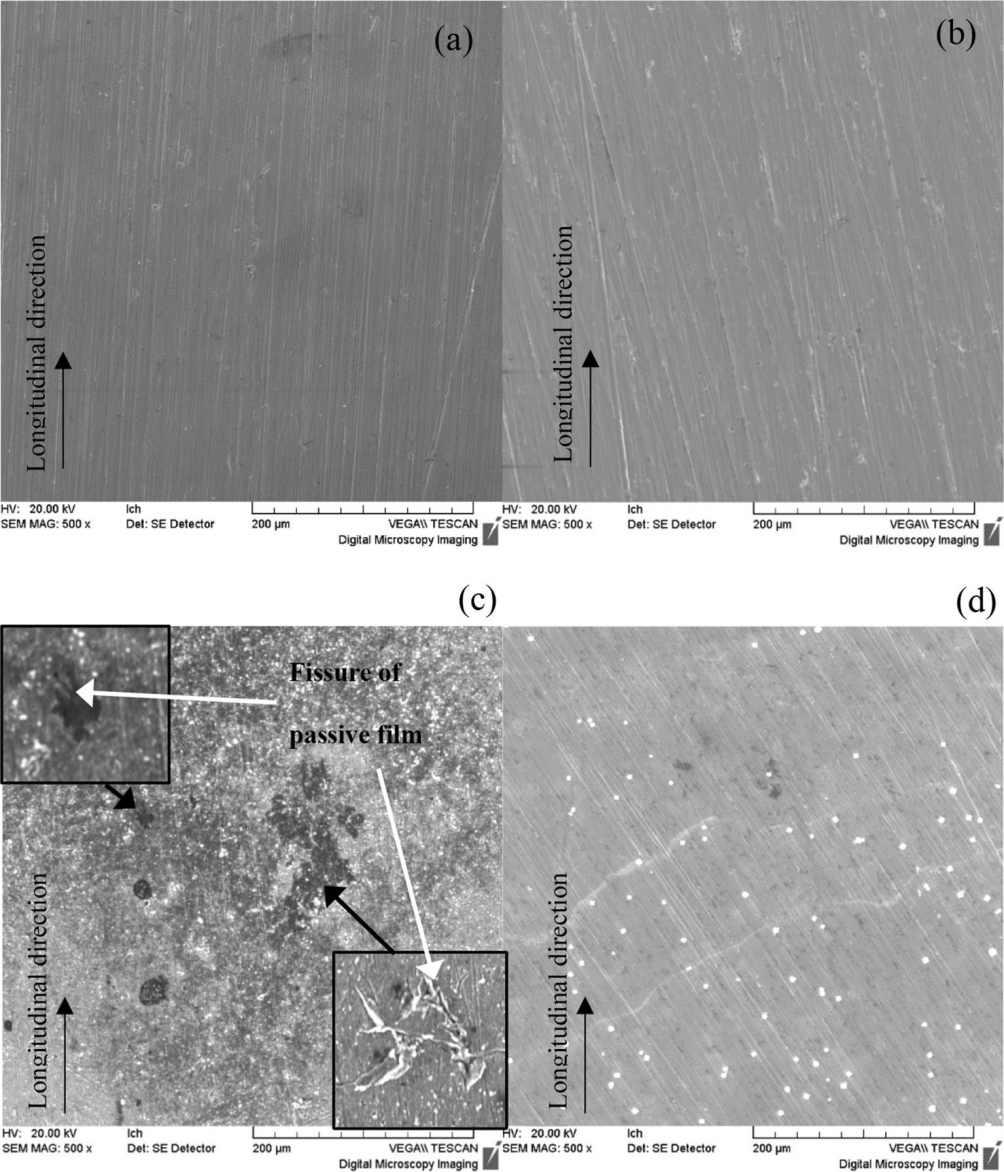
SEM images of surface morphology of the Ti–6Al–4V alloy (**a**) before the test and (**c**) after the test, and the surface morphology of the Ti–6Al–4V-0.11Ru alloy (**b**) before the test and (**d**) after the test.

To compare the surface morphology of passivation films on the Ti–6Al–4V-0.11Ru alloy and the Ti–6Al–4V alloy under high-temperature service conditions, corrosion and crevice tests were conducted in a high-temperature, high-pressure autoclave under test Condition D. After 72 h of immersion, the surface morphology of the two titanium alloys was observed by SEM and is shown in Fig. 9. It was revealed that there were no significant differences between the two alloys before the test; however, remarkable local corrosion was observed on the surface of the Ti–6Al–4V alloy with several deep pits nearby. These pits were approximately 10–100 μm in diameter, and obvious microcracks were found around and at the bottom of the pits, as shown by the magnified images in Fig. 9c. It seems evident that the failure of the passive film on the surface of the Ti–6Al–4V alloy occurred from two main processes: the dissolution of the passive film and the initiation of microcracks, which was in good agreement with the research of Li et al.^25^ In contrast, polish marks were still seen on the surface of the Ti-6Al-4V-0.11Ru alloy without obvious corrosion traces, indicating that the Ru-containing titanium alloy material had better corrosion resistance under this test condition.

**Figure 10:**
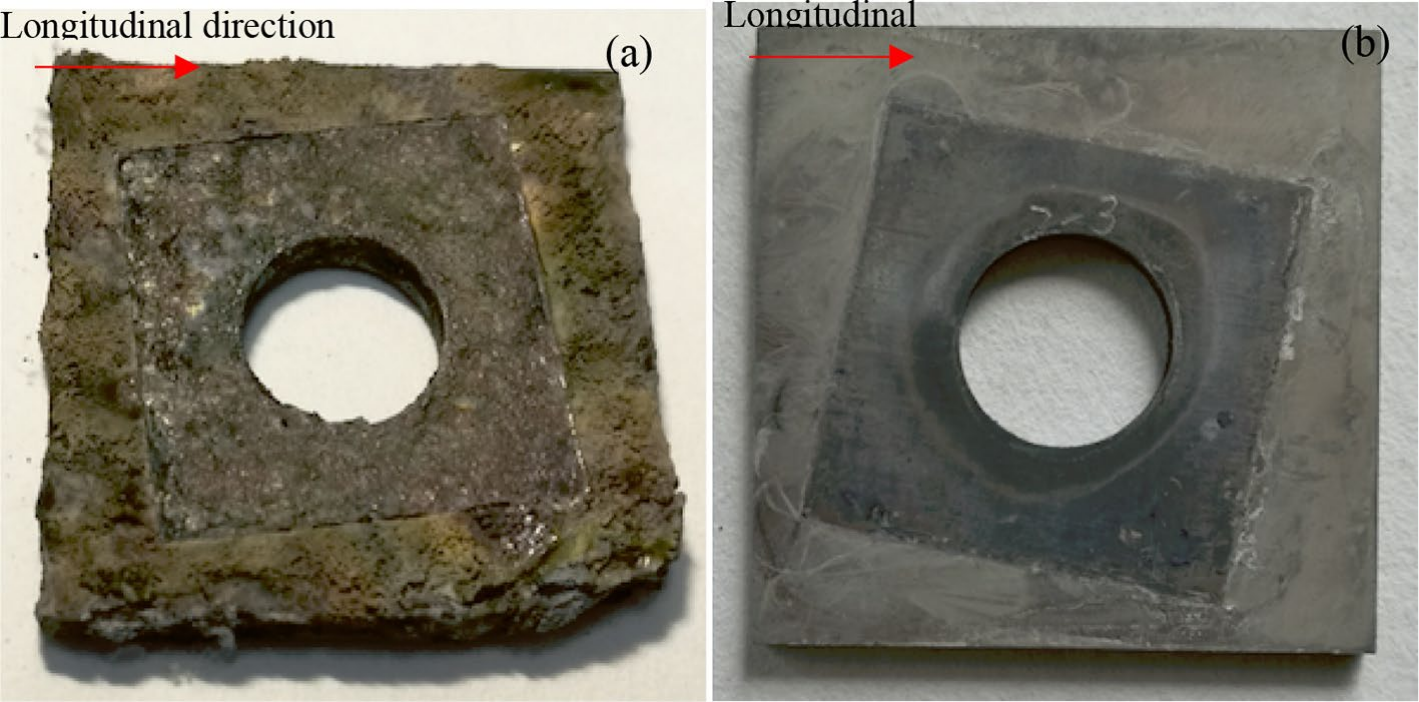
Surface morphologies of different titanium alloys after the crevice corrosion test: (**a**) Ti-6Al-4V, (**b**) Ti-6Al-4V-0.11Ru.

Figure 10 illustrates the surface morphology of the two tested alloys after the crevice corrosion test. The surface morphologies of the two titanium alloys were distinguishable after the crevice corrosion test. No obvious corrosion traces could be found on the surface of the Ti-6Al-4V-0.11Ru alloy, while many corrosion products were produced on the surface of the Ti-6Al-4V alloy, and there were multiple instances of cracking and spalling around the edges of the sample. The EDS results showed that the main corrosion products were alumina and titanium oxide.

**Figure 11:**
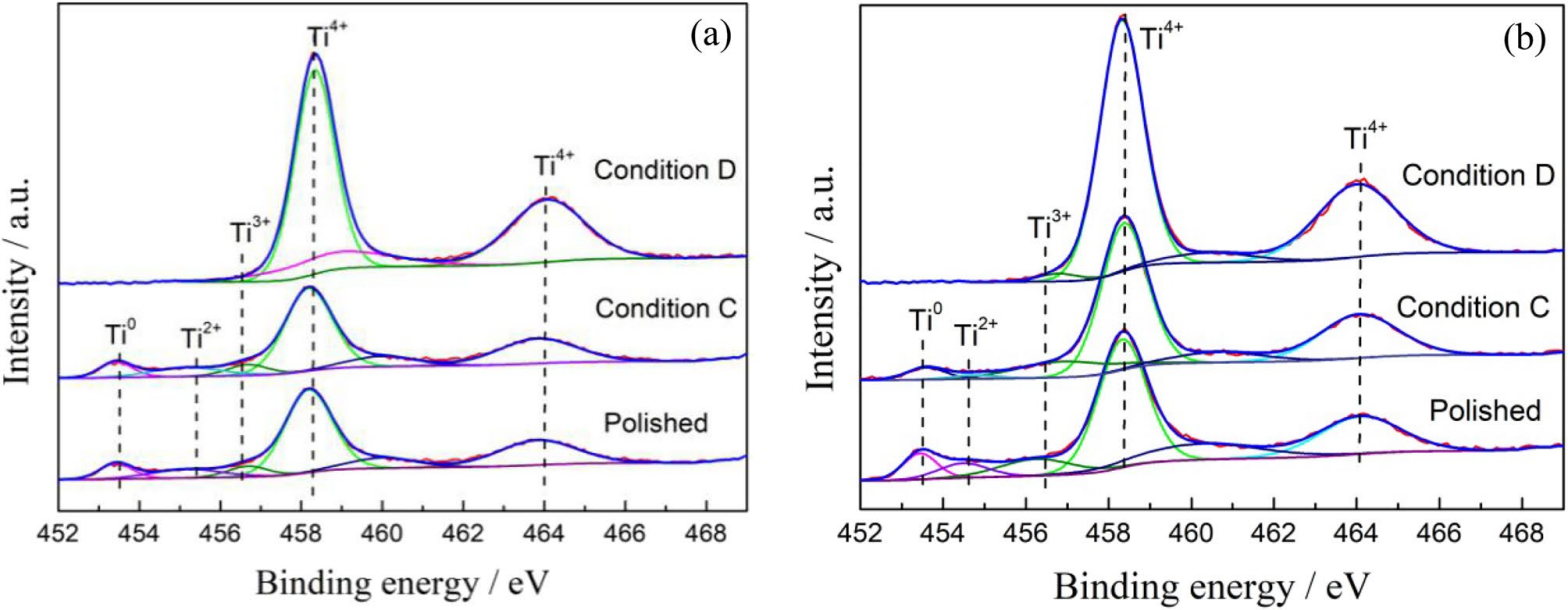
Deconvoluted Ti_2p_ XPS spectra for (**a**) Ti-6Al-4V and (**b**) Ti-6Al-4V-0.11Ru under different conditions.

**Figure 12:**
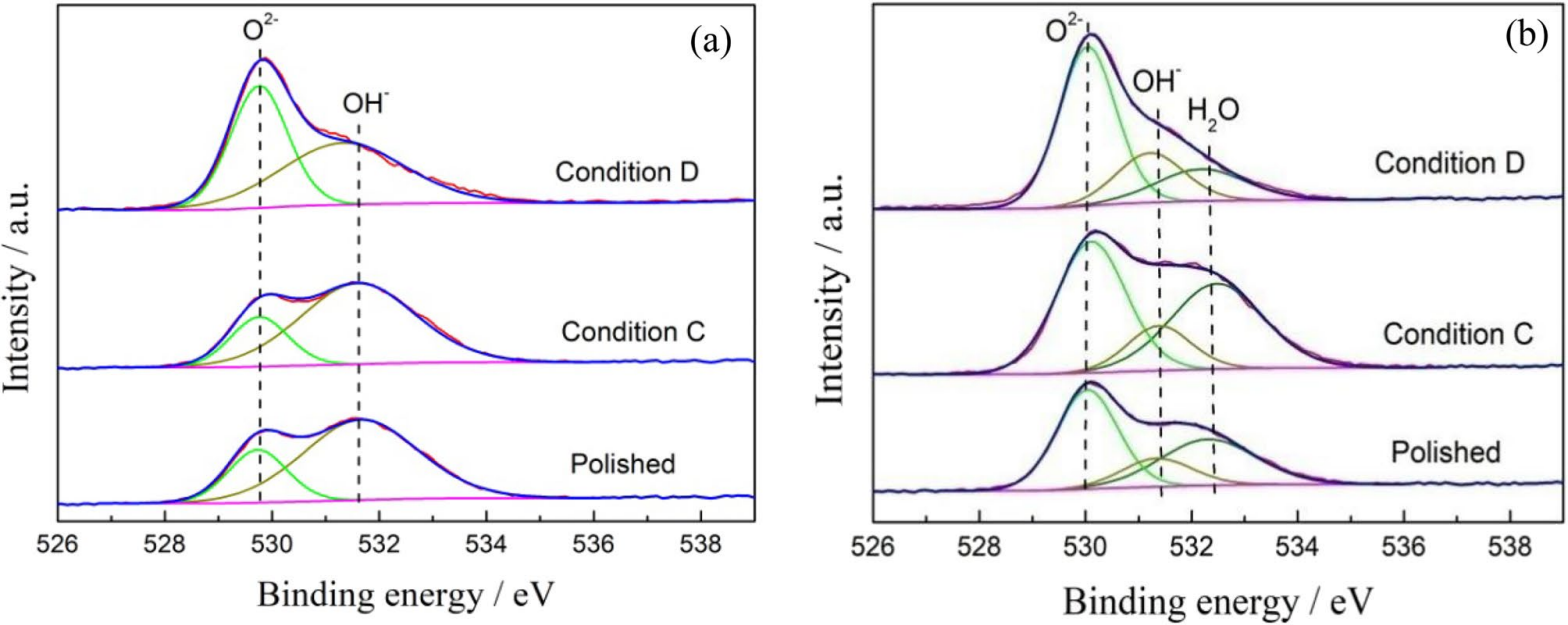
Deconvoluted O_1s_ XPS spectra for (**a**) Ti-6Al-4V and (**b**) Ti-6Al-4V-0.11Ru under different conditions.

The samples of Ti-6Al-4V and Ti-6Al-4V-0.11Ru alloys after immersion in Conditions C and D and in air were selected to detect the surface chemical composition by XPS analysis. The binding energies of titanium, oxygen, aluminium, vanadium and ruthenium were determined according to the NIST XPS database by Avantage software. Figures 11 and 12 show deconvoluted high-resolution Ti_2p_ and O_1s_ XPS spectra for the two tested titanium alloys under different conditions. The concentrations of Ti and O species are summarized in Table 3. It was revealed that tetravalent titanium oxides were prominent on the surface film of both tested alloys with doublets at approximately 458.4 eV and 464.2 eV under all conditions, which indicated that the passive films of the two alloys were mainly composed of TiO_2_^40^. At the same time, moderate amounts of trivalent titanium and bivalent titanium existed in the surface films of the two tested alloys after polishing under Condition C, which represented the metastable oxides of Ti_2_O_3_ and ${{\mathrm{TiO}}_{2}}^{2-}$^41,42^ according to the XPS spectra results of O_1s_ in Fig. 12. It was also noted that there was little difference in the suboxide content on the surface film of the two alloys after polishing under Condition C. However, after 72 h of immersion under Condition D, the chemical composition on the surface film of the two titanium alloys was significantly different; no obvious peak of suboxide was detected on the surface film of the Ti-6Al-4V-0.11Ru alloy, and the oxide film of the Ti-6Al-4V-0.11Ru alloy consisted solely of tetravalent titanium oxides TiO_2_. In contrast, the contents of unstable oxides such as Ti_2_O_3_ in the surface film of the Ti-6Al-4V alloy increased significantly, and the contents of tetravalent titanium oxides were much lower than that of the Ti-6Al-4V-0.11Ru alloy, which made a dramatic difference between the passive film structures of the two titanium alloys.

**Table 3:** Element concentration (at.%) of Ti and O species on the surface of Ti-6Al-4V and Ti-6Al-4V-0.11Ru alloys under three conditions.

Materials	Conditions	Concentration/at.%						
		Ti^4+^	Ti^3+^	Ti^2+^	Ti^0^	O^2-^	OH^−^	H_2_O
Ti-6Al-4V	Polished	72.70	8.02	9.75	9.53	25.04	74.96	–
	C	76.78	10.04	7.16	6.02	32.91	67.09	–
	D	80.38	19.62	–	–	73.99	26.01	–
Ti-6Al-4V-0.11Ru	Polished	73.12	11.22	6.54	9.12	50.01	17.87	32.13
	C	77.43	10.87	7.76	3.94	49.18	12.64	38.81
	D	95.92	1.77	2.32	–	56.65	19.30	24.05

**Figure 13:**
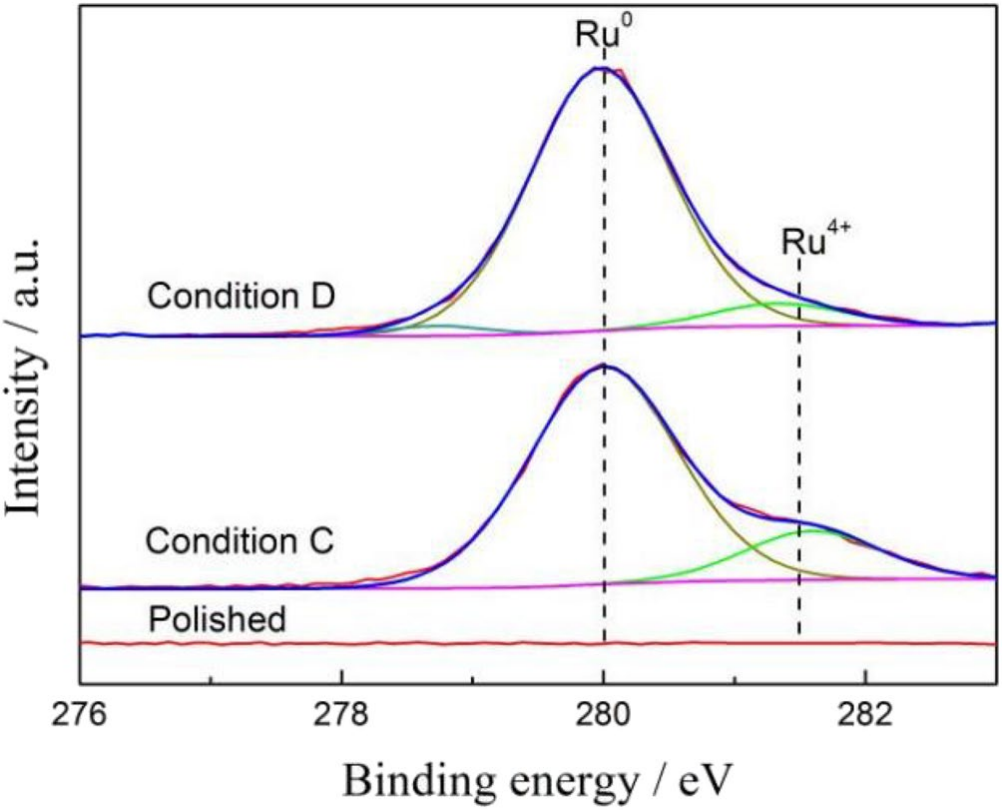
Deconvoluted Ru_3d2/5_ XPS spectra for Ti-6Al-4V-0.11Ru alloys under different test conditions.

Figure 13 illustrates the deconvoluted high-resolution XPS spectra of Ru_3d_ on the surface film for the Ti-6Al-4V-0.11Ru alloy under different test conditions. There was no obvious peak of ruthenium detected after polishing, but the XPS spectrum of Ru_3d_ changed dramatically with increasing temperature under immersion Conditions C and D. Interestingly, the binding energy of the Ru_3d_ peak was generally close to 280.0 eV, which meant that metallic ruthenium existed in the surface film of the Ti-6Al-4V-0.11Ru alloy^43^. Comparing the elemental concentration of ruthenium on the surface films of the Ti-6Al-4V-0.11Ru alloy under the two immersion conditions, the content of metallic ruthenium increased from 82.99 to 90.24% with increasing temperature, accompanied by a decrease in the content of tetravalent ruthenium oxide RuO_2_.

## Discussion

**Figure 14:**
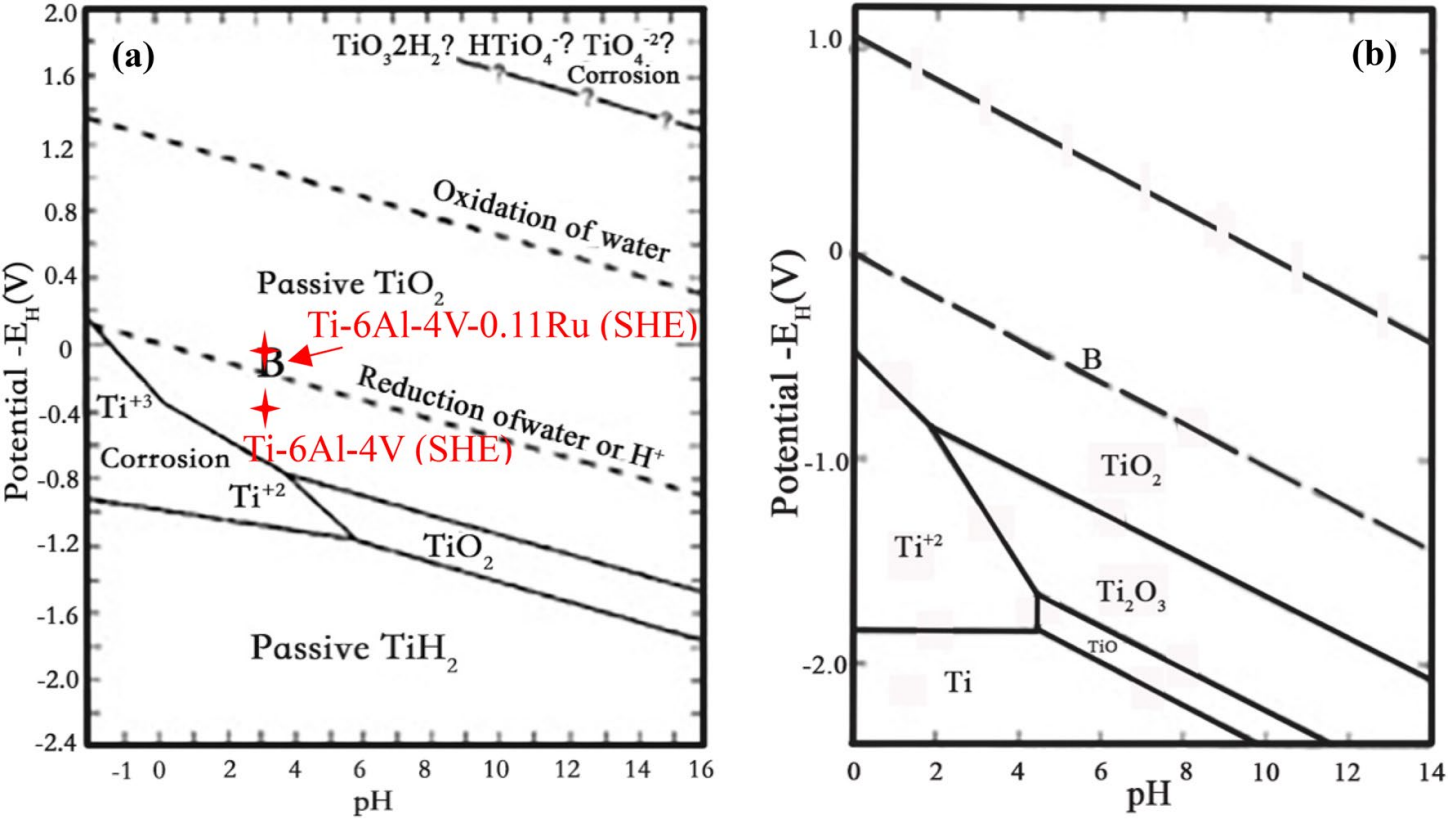
Pourbaix E–pH diagram for titanium in water: (**a**) 23 °C^48^and (**b**) 250 °C^49^.

Many studies have been conducted that have shown that the excellent corrosion resistance of titanium and titanium alloys in a wide range of environments is due to the formation of a dense and protective oxide film on the surface of the metal^44–47^, according to the Pourbaix E–pH diagram for titanium in water^48,49^, as shown in Fig. 14. When the pH value of the solution is greater than 6 at ambient temperature, the passivation state of the oxide film on the surface can be maintained, and the reduction reaction line of water or H^+^ is in the zone of passive TiO_2_, which means that the formation of passive films on the surface of titanium is sustainable in water. If the pH value of the solution is less than 5, whether the titanium or titanium alloy can keep the passivation film insoluble depends on the surface potential of the different alloys. From previous electrochemical performance analysis results, the final OCP values of the Ti-6Al-4V alloy and Ti-6Al-4V-0.11Ru alloy stabilized at approximately − 632 mV_SCE_ and − 152 mV_SCE_, respectively, at 23 °C and pH = 3, which were all in the zone of passive TiO_2_. Table 3 shows that the corrosion current on the surface of the two tested titanium alloys was relatively low, and all of the alloys had good corrosion resistance.

Under the influence of high temperature, high pressure, high corrosion and other factors in harsh oil and gas exploration conditions^4^, the titanium alloy material was gradually oxidized by dissolved oxygen into blunt oxide in narrow crevices, such as the large number of tiny joint crevices between the threaded connections of tubular goods, and further formed complexes with a high content of chlorine ions in the solution. When the oxygen in the gap space was gradually exhausted and the dissolution rate of the passive film increased, the number of dissolved titanium ions increased. When the number of corrosion products, such as titanium ions, in the gap reached a certain concentration, the following self-dissolution and oxidation reaction occurred on the surface film of titanium alloys as the cathode:

6 $$\mathrm{Ti}\to {\mathrm{Ti}}^{3+}+3{\mathrm{e}}^{-}$$

and

7 $${\mathrm{Ti}}^{3+}+{3\mathrm{H}}_{2}\mathrm{O}\to {\mathrm{Ti}(\mathrm{OH})}_{3}+3{\mathrm{H}}^{+}$$

8 $${\mathrm{Ti}}^{3+}+{2\mathrm{H}}_{2}\mathrm{O}\to {\mathrm{TiO}}_{2}+4{\mathrm{H}}^{+} +\mathrm{e}$$

The pH value in the tubular gap space decreased rapidly^50^. According to Fig. 14, with increasing temperature, the corrosion region of titanium in the Pourbaix E–pH diagram gradually expanded, and the passive titanium alloy films dissolved to different degrees according to the surface potential. When the pH value of the test solution decreased to 1.5 and the temperature increased, the steady OCPs and the corrosion potential (E_corr_) of the Ti-6Al-4V alloy shifted negatively, resulting in a significant increase in the corrosion current and a decrease in the passivation film resistance.

The corrosion current and corrosion resistance of the titanium alloy under different service conditions are a comprehensive reflection of the local fine structure, composition, electron structure, thickness and chemical properties of the surface passive film. Various titanium oxides coexist in the passive film of the titanium surface, such as TiO, Ti_2_O_3_ and TiO_2_. According to Hanawa et al.^51^, the surface film of titanium alloys is oxidized into TiO initially and then quickly changes into Ti_2_O_3_ due to the thermodynamic stability. Furthermore, Ti_2_O_3_ is oxidized into hydroxide and dehydrated to stable tetravalent titanium oxides TiO_2_, as follows:

9 $${\mathrm{Ti}}_{2}{\mathrm{O}}_{3}+3{\mathrm{H}}_{2}\mathrm{O}\to {2\mathrm{TiO}\left(\mathrm{OH}\right)}_{2}+2{\mathrm{H}}^{+}+2{\mathrm{e}}^{-}$$

10 $${2\mathrm{TiO}\left(\mathrm{OH}\right)}_{2}\to 2{\mathrm{TiO}}_{2}+2{\mathrm{H}}_{2}\mathrm{O}$$

Wang et al. investigated the local fine structure of a titanium passive film and revealed that the passivation of the thin film greatly depended on the surface potential and the O^2−^ component, and the ratio of TiO, Ti_2_O_3_ and TiO_2_ changed significantly with the surface potential^52,53^. Ohtuska et al. found that the passive film of titanium alloys consisted of the tetravalent titanium oxide TiO_2_ with an amorphous or low crystallinity structure at a film formation potential of approximately 7 V_SCE_^54^ because the tetravalent titanium in TiO_2_ is the maximum valence state that cannot be further oxidized, so it is more stable than the metastable oxides of titanium. Figure 14 shows that the titanium surface would fall in the corrosion region of the titanium Pourbaix E–pH diagram under a harsh oil and gas exploration environment. The stability of the surface film is related to the competition of dissolution propensity and passivation ability. The chemical dissolution of tetravalent titanium oxide is thermodynamically spontaneous^25,55^, and the electronic structure and chemical structure of the passive film will be changed through the oxidation reaction because of the change in the surface potentials^56,57^. The reduction reaction of TiO_2_ will occur on the surface films of titanium alloys as follows:

11 $$2{\mathrm{TiO}}_{2}+{2\mathrm{H}}^{+}+2{\mathrm{e}}^{-} \to {\mathrm{Ti}}_{2}{\mathrm{O}}_{3}+{\mathrm{H}}_{2}\mathrm{O}$$

Trivalent titanium and bivalent titanium are metastable oxides of titanium that are less stable than tetravalent titanium. These metastable oxides act as defects in the passive film, resulting in the dissolution of the passive film and an obvious increase in the current density of the alloy surface under the test conditions.

The results of XPS analysis show that the content of metastable titanium oxides in the passivation film on the surface of the Ti-6Al-4V alloy increased significantly due to the temperature rise and pH drop under the service conditions, resulting in a remarkable decrease in the thickness and stability of the passivation film. Due to the decrease in the protective ability of the surface passivation film, severe local corrosion and crevice corrosion occurred on the surface of the Ti–6Al–4V alloy, as shown in Figs. 9 and 11. Therefore, the dissolution of the unstable passive film led to an increase in the corrosion current density.

When adding trace amounts of ruthenium to the Ti-6Al-4V alloy, the corrosion potential of the passive film on the Ti-6Al-4V-0.11Ru alloy surface was shifted significantly in the noble direction because of the high surface potential of the ruthenium element. A. Biesiekierski found that minimal Ru additions significantly altered the corrosion potential, yielding a 0.3 V shift in the noble direction over the Ru-free controls^58,59^. F. King investigated ruthenium’s role in the titanium alloy in Hank’s balanced salt solution, and the results revealed that Ru could catalyse the reduction of H^+^ on the surface of alloy and prevent the acidification of crevice environments^60^. As is apparent from the XPS results in Fig. 16, no ruthenium element was found on the surface of the passivation film before the test; however, metallic ruthenium and tetravalent ruthenium oxide RuO_2_ were obviously present in the passivation film on the surface of the Ti-6Al-4V-0.11Ru alloy under the test conditions. The increase in the content of metallic ruthenium was due to the following reaction:

12 $$\frac{3}{2}{\mathrm{RuO}}_{2}\to {\mathrm{RuO}}_{3}+\frac{1}{2}{\mathrm{Ru}}^{0}$$

RuO_3_ vapourised at low temperatures so that the ruthenium element in the surface film was mainly composed of metallic ruthenium and RuO_2_^61^.

**Figure 15:**
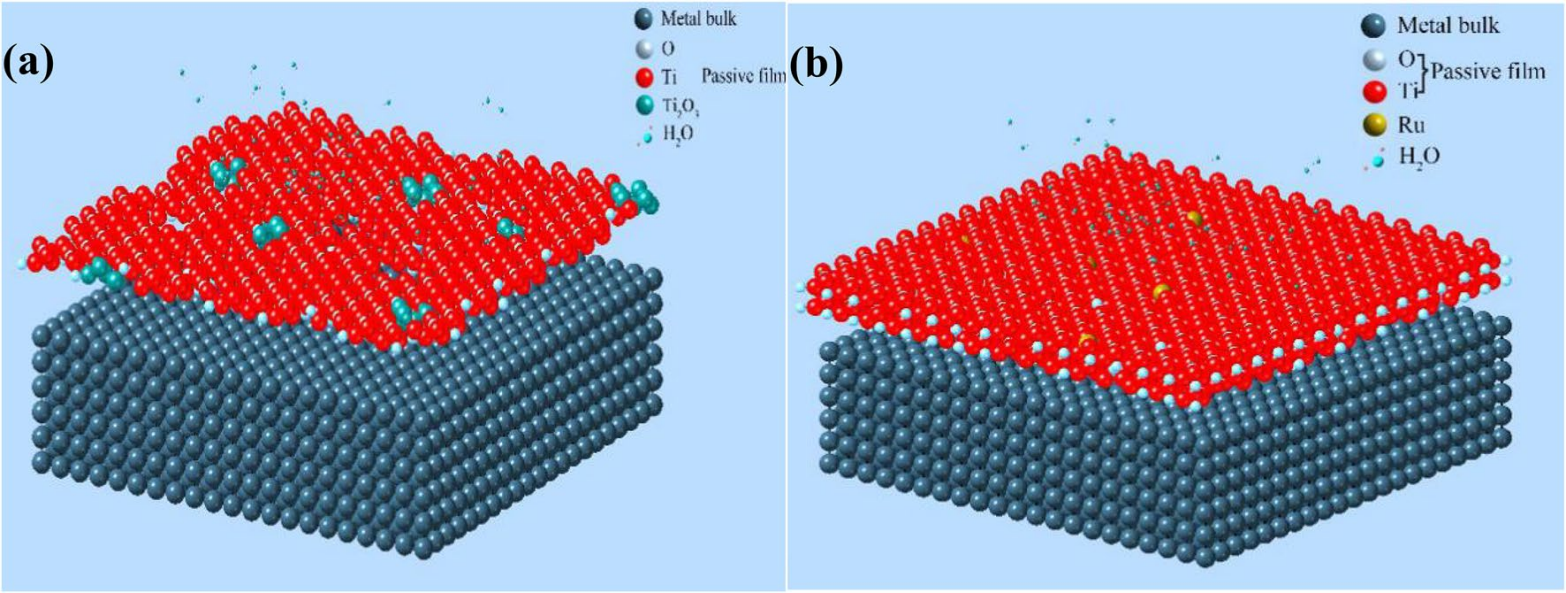
Schematic diagram of the influence model of Ru on the passive film thickness and structure of Ti-6Al-4V alloy (**a**) without Ru and (**b**) Ru-containing (Note: the locations of O, Ru and H_2_O are arbitrary).

The influence of Ru on the characteristics and passivation mechanism of the passive film on the surface of the Ti-6Al-4V alloy can be summarized by the model in Fig. 15, the locations of O, Ru and H2O are arbitrary and just for illustration purpose. When the titanium alloy OCTG were tested in the harsh oil and gas exploration conditions, the passivation film on the surface of the Ti-6Al-4V alloy was damaged and dissolved due to the existence of many metastable titanium oxides and the lower surface potential, causing local corrosion and crevice corrosion on the alloy, as shown in Fig. 15a. However, when adding trace amounts of ruthenium elements to the alloy, the electronic and chemical structure of the passive film changed dramatically. As the top layer of the passivation film on the surface of the Ti-6Al-4V-0.11Ru alloy was dissolved under harsh oil and gas exploration conditions, the metallic ruthenium and RuO_2_ in the passive film increased the surface potential of the top layer, resulting in an increase in passivation ability and a decrease in the content of metastable oxides in the passivation film, improving the thickness and passivation ability of the passivation film and the stability, repairability and corrosion resistance of the Ti-6Al-4V-0.11Ru alloy^62^, as shown in Fig. 15b.

**Figure 16:**
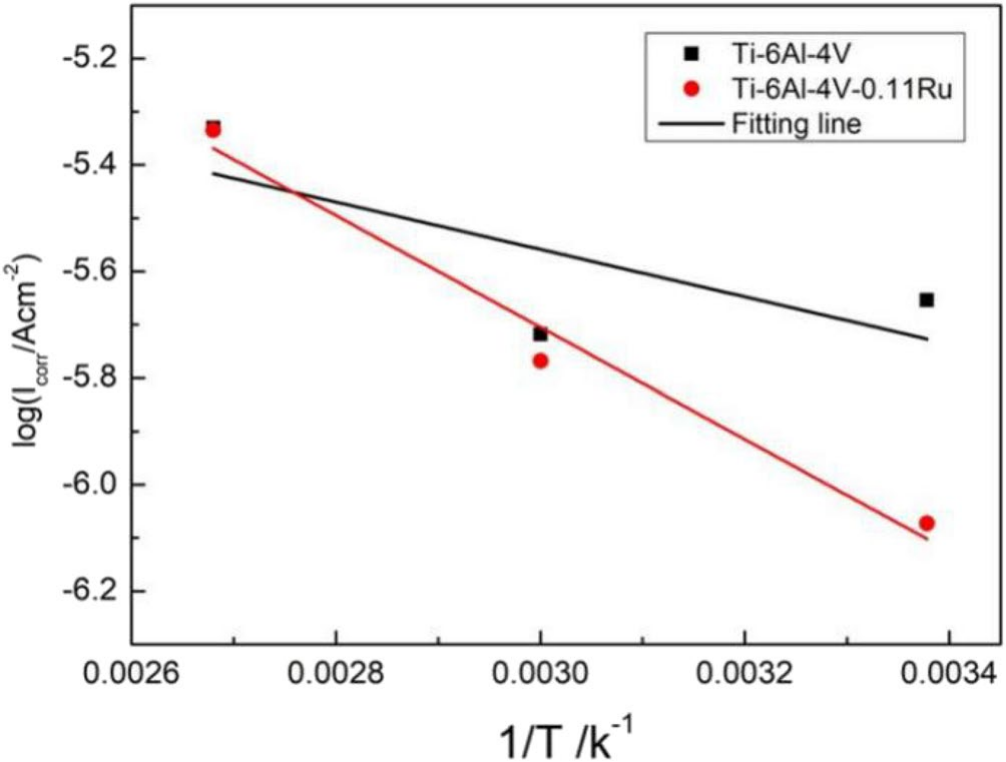
The Arrhenius plots of Ti-6Al-4V and Ti-6Al-4V-0.11Ru alloys with different temperatures.

The relationship between the corrosion resistance and temperature was examined by the Arrhenius equation^63^:

13 $$\frac{dlog{I}_{corr}}{d(1/T)}=-\frac{{E}_{a}}{2.303R}$$

where ${E}_{a}$ is the apparent activation energy of the corrosion process, T is the absolute temperature and R is the gas constant. The Arrhenius plots of Ti-6Al-4V and Ti-6Al-4V-0.11Ru alloys are presented in Fig. 18, and the apparent activation energies are 8.52 kJ mol^−1^ and 20.09 kJ mol^−1^ for Ti-6Al-4V and Ti-6Al-4V-0.11Ru, respectively. These results provided further evidence for the better corrosion resistance of the Ti-6Al-4V-0.11Ru alloy than the Ti-6Al-4V alloy (Fig. 16).

## Conclusions

In the present study, the influence of Ru on the corrosion behaviour and structural characteristics of passive films on Ti-6Al-4V alloy in harsh oil and gas exploration conditions was investigated by electrochemical techniques, XPS, SEM and corrosion simulation tests. The following conclusions could be drawn from this work:

1. The passive films were rapidly formed and stabilized on the surfaces of all titanium alloys under oil and gas exploration conditions. The stability of the passive film on the Ti-6Al-4V alloy surface decreased with increasing temperature, and the thermodynamic stability of the Ti-6Al-4V-0.11Ru alloy was better than that of Ti-6Al-4V.
2. The Ti-6Al-4V alloy with the addition of ruthenium had a higher corrosion potential and lower corrosion current density under all test conditions, as well as a better surface repassivation ability.
3. The EIS results indicated that dense passive films formed on the surface of the tested alloys and became thinner when the temperature increased. From the analysis results of EIS and passivation film thickness, the Ti-6Al-4V-0.11Ru alloy exhibited a significantly higher corrosion resistance than the Ti-6Al-4V alloy under all experimental conditions.
4. The passive films of the two alloys were mainly composed of TiO_2_, and the contents of metastable oxides such as Ti_2_O_3_ and ${{\mathrm{TiO}}_{2}}^{2-}$ in the passive film of the Ti-6Al-4V alloy increased significantly with increasing temperature, while the passive film of the Ti-6Al-4V-0.11Ru alloy consisted solely of tetravalent titanium oxide TiO_2_, and metallic ruthenium and tetravalent ruthenium oxide RuO_2_, as demonstrated by XPS.
5. With the addition of ruthenium, the electronic and chemical structure of the passive film changed significantly, and the surface potential of the top passive film on the surface of the Ti-6Al-4V-0.11Ru alloy was raised because the metallic ruthenium and RuO_2_ in the passive film increased the passivation ability and decreased the content of metastable oxides in the passivation film, which increased the stability, corrosion resistance, repairability and thickness of the passive film on the surface of the Ti-6Al-4V-0.11Ru alloy being better than those qualities of the Ti-6Al-4V alloy. These results were also proven by corrosion simulation tests.

## Methods

### Materials preparation

**Figure 17:**
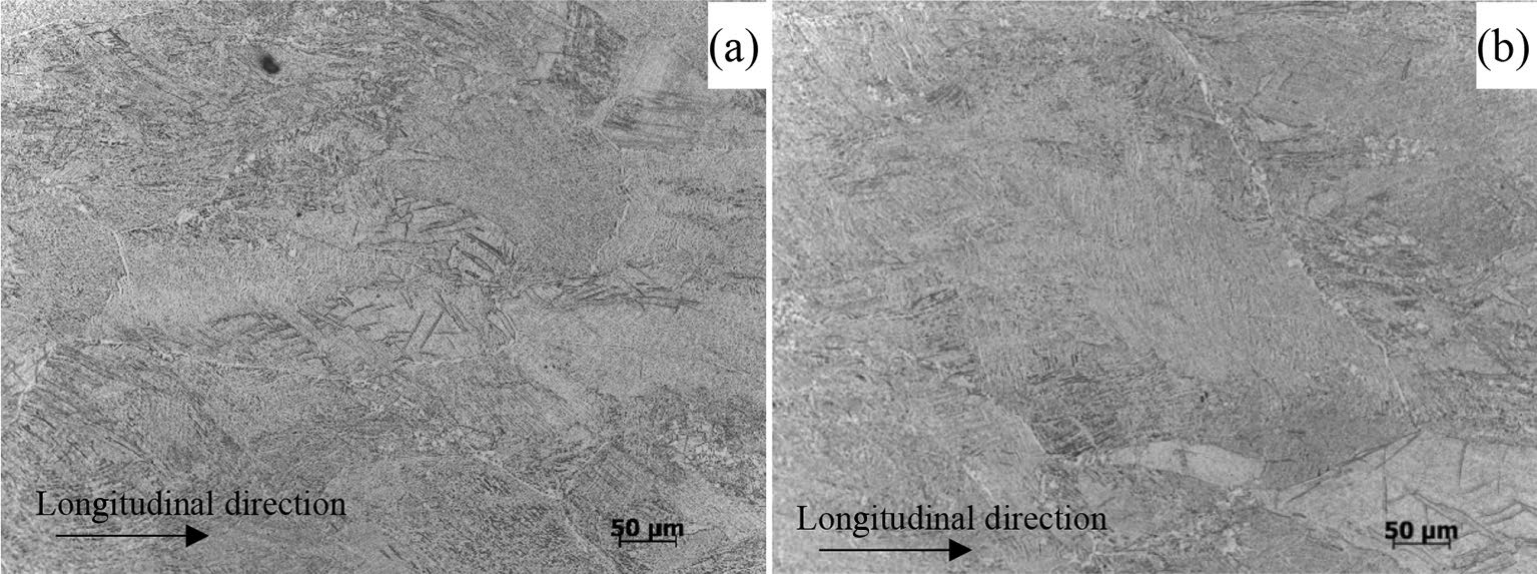
Microstructures of titanium alloy samples: (**a**) Ti-6Al-4V-0.11Ru and (**b**) Ti-6Al-4V.

The experimental titanium alloys were cut from a tube that was 88.9 mm in diameter and 10 mm in thickness supplied by the CNPC Tubular Goods Research Institute of China. The composition and microstructure samples were taken from a longitudinal tube 15 mm × 15 mm × 10 mm. After undergoing wet grinding and diamond paste polishing with pastes of 6 µm and 1 µm, the chemical compositions of the prepared samples were analyzed using a Thermo iCAP 6300 plasma emission spectrometer, and the chemical compositions (in wt. %) of all tested samples are presented in Table 4. The microstructures of the samples were analyzed using MEF3A and MEF4M metallographic microscopes. The metallographic structures of the two alloys are both Widmanstatten structures, as shown in Fig. 17.

**Table 4:** Chemical composition of titanium alloy samples used in the tests (wt. %).

Samples	Al	V	Ru	O	C	H	N	Ti
Ti-6Al-4V-0.11Ru	5.69	3.97	0.11	0.07	0.0082	0.0035	0.007	Bal
Ti-6Al-4V	5.89	4.00	–	0.074	0.0092	0.0055	0.008	Bal

### Working electrolyte conditions

For electrochemical measurements and corrosion resistance evaluations, the typical oil and gas exploration conditions of an oil field in western China were selected. Considering the change in the working environment at different stages in the process of oil and gas exploration, the influences of different pH values and temperature conditions in the simulated medium environment were added, as well as different working electrolyte conditions. All of these variables are listed in Table 5. CO_2_ and H_2_S with 99.9% purity were injected into the high-temperature autoclave through a booster pump to establish the required partial pressure, and the total pressure was established by injecting pure N_2_.

**Table 5:** Test conditions of electrochemical measurements and corrosion resistance evaluation.

Test conditions	pH	Temperature/˚C	Total pressure/MPa	H_2_S partial pressure/MPa	CO_2_ partial pressure/MPa	Ionic composition/mg·L^−1^
Condition A	3	23	12 (for all test conditions)	1.15–1.19 (for all test conditions)	6 (for all test conditions)	HCO_3_^−^/189, Cl^−^/128,000, SO_4_^2−^/430, Ca^2+^/8310, Mg^2+^/561, K^+^/6620, Na^+^/76,500 (for all test conditions)
Condition B	1.5	23				
Condition C	1.5	60				
Condition D	1.5	100				

### Electrochemical measurement

Electrochemical test specimens were cut from the tube near its inner face. The samples were all in the form of discs with 15 mm diameters and 3 mm thicknesses and sealed with epoxy resin in a special ring. The exposed area of the samples were all with an area of 1.76 cm^2^. All samples were polished by silicon carbide papers from 240 to 1200 grit and ultrasonically cleaned with distilled water, acetone and ethanol.

The electrochemical tests were carried out in a conventional water-jacketed three-electrode electrochemical cell with a Pt plate counter electrode and an Ag/AgCl reference electrode. A jacketed salt bridge with an Ag/AgCl electrode was used to cool it to ambient temperature, and the saturated KBr solution was filled in salt bridge with Ag/AgCl. Electrochemical measurements were performed with an AMETEK 273A electrochemical workstation. Note that the potentials in the text refer to the SCE scale at room temperature. To stabilize the electrode/solution interface before testing, all samples were pretreated by -1.2 V_SCE_ for 5 min to remove the oxide from the sample surface and then immersed in test solution in a high-temperature, high-pressure autoclave for 72 h. After immersion, time-dependent open-circuit potential (OCP) tests were carried out on all samples for 24 h to reach steady state passivation. The electrochemical impedance spectroscopy (EIS) measurements were conducted after the OCP tests. The scanning frequency range was 10^5^–0.01 Hz at 10 points per decade, and a ± 10 mV signal amplitude with a sine wave form was applied to the open-circuit potentials. After EIS tests, potentiodynamic polarization (PDP) experiments were performed from − 500 mV vs OCP to 1600 mV_SCE_ with a scan rate of 0.5 mV/s. Three tests were repeated for each material and condition to obtain stable results. All the data were interpreted on the basis of equivalent electrical circuits and analyzed by Zview software.

### Corrosion simulation test

**Figure 18:**
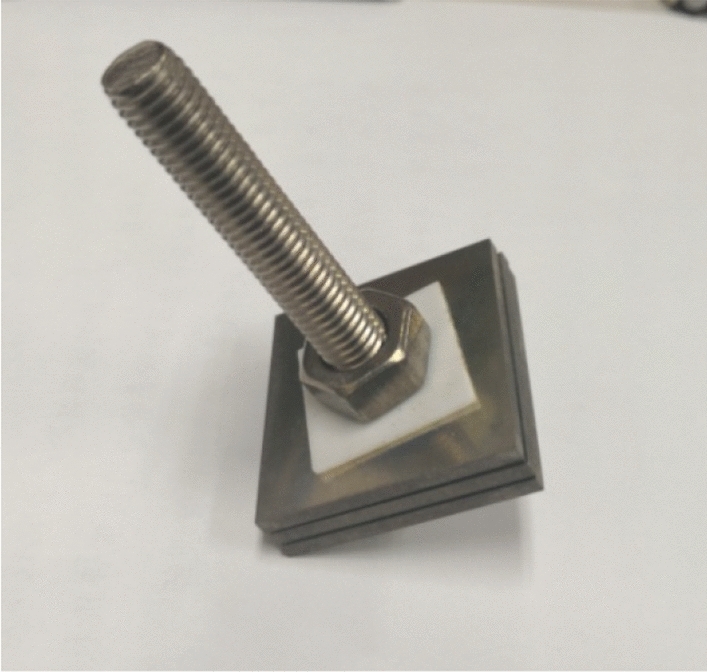
Crevice corrosion specimens of titanium alloys used in tests.

In the uniform corrosion experiment, hanging samples with a size of 40 mm × 10 mm × 3 mm were prepared from a longitudinal pipe and polished by silicon carbide papers from 240 to 1200 grit until the surface roughness was less than 1.6 μm. Crevice corrosion specimens were taken from the tube body in a square piece with dimensions of 38 mm × 38 mm × 3 mm. After polishing with 1000-grit sandpaper, a Φ 10 mm-round hole was drilled in the centre of each specimen. A 0.3 mm × 15 mm × 15 mm PTFE gasket was used to form a gap between two square plates. All the crevice corrosion specimens were connected to each other by titanium alloy bolts of the same material to avoid galvanic coupling between the bolts and the sample, so the Ti-6Al-4V-0.11Ru alloy samples were paired with Ti-6Al-4V-0.11Ru alloy bolts and the Ti-6Al-4V samples were paired with Ti-6Al-4V alloy bolts. A torque wrench of 86 cm•kg was applied uniformly to tighten the test piece string to form the crevice corrosion samples according to the same test^64^, as shown in Fig. 18.

The solution of working conditions D was prepared according to Table 5, and corrosion samples were loaded into a high-temperature, high-pressure autoclave manufactured by Cortest Company. Before the corrosion test, high purity nitrogen was flowed through the autoclave for more than 10 h for deoxygenation. Then, the samples were loaded, and the autoclave was sealed. High purity nitrogen was continuously injected for further deoxygenation, and medium gas was injected. When the temperature in the autoclave was raised to the required temperature, the test timing was started.

### XPS analysis and microstructure observation

The surface chemical compositions of the original alloy samples and immersed samples were analyzed and compared with XPS using a K-alpha XPS spectrometer with a monochromatic Al K_α_ X-ray source and a take-off angle of 90°. The pass energy was 50 eV with an energy step size of 0.1 eV for the high-resolution scan, and the area of the beam spot was 400 μm. The standard binding energies of titanium, oxygen, aluminium, vanadium and ruthenium were determined according to the NIST XPS database.

The surface morphology of the samples after the corrosion and immersion tests was observed and analysed by a TESCAN VEGA-II scanning electron microscope (SEM) and the OXFORD-INCA350 energy dispersive spectrometer (EDS).

## Data Availability

The datasets generated and analyzed during the current research period are not disclosed due to China National Petroleum Corporation (CNPC)'s information protection and confidentiality regulations, but could be available at the reasonable request of the corresponding authors and after approval by the CNPC and TGRI technology and information management departments.
